# Nanodiamonds as Possible Tools for Improved Management of Bladder Cancer and Bacterial Cystitis

**DOI:** 10.3390/ijms23158183

**Published:** 2022-07-25

**Authors:** Daša Zupančič, Peter Veranič

**Affiliations:** Institute of Cell Biology, Faculty of Medicine, University of Ljubljana, Vrazov Trg 2, 1000 Ljubljana, Slovenia; dasa.zupancic@mf.uni-lj.si

**Keywords:** nanodiamonds, functionalisation, urinary bladder, bladder cancer, bacterial cystitis

## Abstract

Nanodiamonds (NDs) are a class of carbon nanomaterials with sizes ranging from a few nm to micrometres. Due to their excellent physical, chemical and optical properties, they have recently attracted much attention in biomedicine. In addition, their exceptional biocompatibility and the possibility of precise surface functionalisation offer promising opportunities for biological applications such as cell labelling and imaging, as well as targeted drug delivery. However, using NDs for selective targeting of desired biomolecules within a complex biological system remains challenging. Urinary bladder cancer and bacterial cystitis are major diseases of the bladder with high incidence and poor treatment options. In this review, we present: (i) the synthesis, properties and functionalisation of NDs; (ii) recent advances in the study of various NDs used for better treatment of bladder cancer and (iii) bacterial cystitis; and (iv) the use of NDs in theranostics of these diseases.

## 1. Introduction

Nanodiamonds (NDs) are carbon-based materials that have attracted considerable interest in biomedical research due to their outstanding properties, particularly their biocompatibility and surface functionalisation capabilities. Nanodiamonds were first synthesised in 1963 by detonation of carbon-based explosives [1,2,3]. For various reasons, including a lack of industrial interest in nanotechnology at the time, the application of these NDs remained unknown and little used until two decades ago. The first report on the application of NDs for fluorescence imaging and demonstration of low toxicity in cells was published by Yu et al. in 2005 [4]. Subsequently, numerous studies have demonstrated the wide-ranging potential of using NDs in various cells and applications, including targeted drug delivery in urothelial cancer cells [5]. In addition to detonation NDs (DNDs), NDs with a fluorescent colour centre, referred to as fluorescent NDs (FDNs), have attracted increasing interest [6]. This is due to their unique optical properties, including exceptional photostability (neither photobleaching nor blinking), near-infrared fluorescence emission (≈650–900 nm), long fluorescence lifetime (≈20 ns) and high fluorescence quantum yield [6]. In addition, FNDs contain nitrogen vacancy centres (NV), which are created by doping nitrogen in synthetic diamonds and provide the exceptional optical properties of FNDs [7]. The origin of the fluorescence lies in the complexes formed by vacancies and impurities in a diamond crystal. Another major advantage of DNDs and FNDs is the wide range of surface modification possibilities that lead to precise surface functionalisation. This review focuses on current information on NDs used for cell labelling, imaging, most likely applications in bladder cancer and bacterial cystitis, and theranostics.

## 2. Synthesis, Properties and Functionalisation of Nanodiamonds

A unique set of outstanding properties of NDs makes them attractive for targeted drug delivery systems, as well as for theranostics and imaging applications. The broad spectrum of key advances includes exceptional biocompatibility, large carrier capacity, versatile surface chemical properties that enhance drug binding and enable sustained drug release, and stable bright fluorescence based on crystallographic defects. Compared to other forms of nanoparticles, NDs also impress with their exceptional physical and chemical stability and inertness, efficacy, biosafety and low cost.

Mass production of NDs began in the early 80s [3], with three main production methods: (i) detonation of carbonaceous explosives (DNDs, which are formed when selected organic compounds are detonated in a controlled manner); (ii) by milling microdiamond powders produced by high-pressure, high-temperature (HPHT) static synthesis at a specific pressure; and (iii) chemical vapour deposition (CVD). These three classes of NDs have different structures and, accordingly, different niche applications [8].

DNDs typically have a round to oval shape and measure about 5 nm in diameter [9]. Their small size and narrow size distribution make them a popular research topic, but unless they are specially purified, they tend to aggregate with each other and form large aggregates. They are also relatively chemically inert, yet reactive enough to allow functionalisation. In addition, their large relative surface area can be used to effectively attach various compounds. All these properties are very advantageous for drug delivery. Due to their structural composition and manufacturing process, DNDs have a graphitic layer on the outside and relatively many impurities and structural defects, which are desirable in labelling applications [10]. Recent developments could make DNDs more useful for labelling. For example, DNDs can be used as carriers for dyes or their surface chemistry can be adjusted to achieve the desired optical properties [9].

Monocrystalline NDs obtained by processing synthetic HPHT diamond are currently commercially available, with the smallest average particle size ranging from 10 nm to up to micrometres. In the HPHT method, large diamonds are first produced, which are later irradiated with high-energy electrons, protons or helium ions to create NV centres. They exist in two charge states, neutral (NV^0^) and negative (NV¯), with ZPL (zero phonon line) emissions of 575 nm and 637 nm, respectively [11]. This is followed by mechanical size reduction and selection to produce FNDs [12,13]. After the generation of vacancies, the irradiated powder is annealed at a temperature above 700 °C to cause the diffusion of vacancies and the formation of NV centres. In fact, the most commonly used annealing parameters for NDs are in the range of 700–800 °C for 1–2 h [13,14]. According to the systematic annealing studies by Havlik et al., the optimum annealing procedure is 900 °C for 1 h [11]. These particles have a blocky shape and contain N as a natural impurity in the form of substitutional nitrogen (N_S_), with a concentration of up to 300 ppm (for HPHT ND type Ib). ND, made from natural diamond type Ia (N_S_ concentration up to 3000 ppm), is also available. High energy particle irradiation of type Ib ND followed by annealing leads to the formation of NV centres with red emission, while the ND of diamond Ia show green luminescence resulting from the formation of N-V-N (H3) centres after irradiation and annealing [8]. Due to their increased brightness, these materials are best suited for optical labelling. Overall, depending on the diamond type, annealing at high temperatures (600 °C and above) leads to a shift of the vacancies towards the nitrogen atoms and to the formation of the colour centres NV (with red fluorescence), H3 (with green fluorescence) or N3 (with blue fluorescence) [14,15]. Diamonds with one of these colour centres are used for photoluminescence (PL) and cathodoluminescence (CL) [16]. NDs that are subjected to intense irradiation without annealing, resulting in amorphisation of the diamond lattice and high light absorption in the infrared, are called INDs and are used for PA imaging [17]. While HPHT NDs usually have sharp edges and a flaky geometry, particles with round shapes have also been produced [9]. These are created from sharp-edged diamond particles by short-term oxidation in molten potassium nitrate [11]. These round-shaped NDs are particularly interesting for applications in cells, as they are internalised into the cells differently than particles with sharp edges [9].

Recently, an attractive alternative method for producing NDs has been developed. This method starts with diamondoid structures, which are small molecular units with a diamond crystal structure. The ND is then grown around this molecule and during this synthesis, foreign atoms can be placed at the desired positions [18,19]. This approach promises the most precise control over the exact position of the defects and could offer the possibility of placing two or more defects at a specific distance from each other. However, the low yields achieved with this method so far have made it difficult to visualise the materials, preventing the full potential of the method from being realised [9]. All in all, the synthesis of NDs with a size of a few nanometres and with specific colour centres is still an urgent goal.

**Figure 1:**
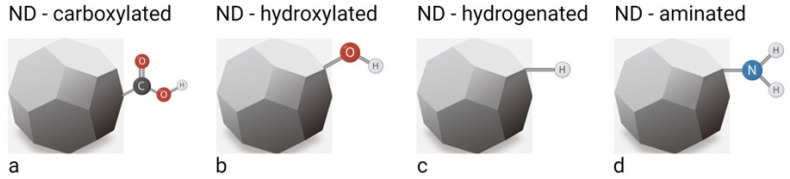
Basic formation of uniform surface constituents on NDs by carboxylation (**a**), hydroxylation (**b**), hydrogenation (**c**) and amination (**d**). The actual surface constituents and their relative abundance are determined by the production methods and subsequent purification steps. The illustration was created by the authors using the BioRender app.

Most of the available DNDs have an oxygenated surface. This is due, for example, to the application of water or ice for cooling in the detonation process, which leads to a reaction with highly reactive hydroxyl species [20]. Only NDs derived from CVD films would initially have a hydrogenated surface, as the feed gas for the deposition of the diamond films consists of a carbon source and a significant amount of hydrogen gas [21]. Regardless of the type of NDs, the formation of uniform surface units prior to performing further reactions on the surface of the NDs is necessary to increase purity, control subsequent reactions on the surface of the NDs and to maximise the influence of the NV centres on the optical properties of the FNDs. Therefore, numerous studies have developed homogenisation processes for NDs, such as carboxylation, hydroxylation, hydrogenation, amination, etc. (Figure 1) [6,22]. These surface modifications are often used as the first step in the functionalisation of NDs. The detailed methodological description of these different processes is described by Hak-Sung Jung et al. in 2021 [6]. Briefly, the formation of carboxyl COOH groups on the surface of NDs is ideally achieved by oxidation treatment with mineral acids and air or ozonated air [23]. Homogenisation of the ND surface with dense OH groups (hydoxylation) is carried out by four different methods: (i) reduction with borane (BH_3_-THF) or lithium aluminium hydride (LiAlH_4_); (ii) by a Fentone reaction (a solution of H_2_O_2_ with iron (II) sulphate (FeSo_4_)); (iii) by a mechanochemical treatment; (iv) by a photochemical reaction [24,25,26,27,28]. Hydrogenation of the ND surface can be achieved by thermal annealing or microwave plasma treatment in a hydrogen atmosphere [23,29,30]. Several approaches have been developed to link NH_2_ groups on the surface of NDs (amination), including ultraviolet (UV) irradiation of hydrogenated diamonds in the presence of chlorine gas, treatment of chlorinated NDs with ammonia at elevated temperatures [31], and treatment of OH groups on the ND surface with (3-aminopropyl)trimethoxysilane (APTES), which results in the formation of an amino group linked to the surface by the APTES molecule [25].

**Figure 2:**
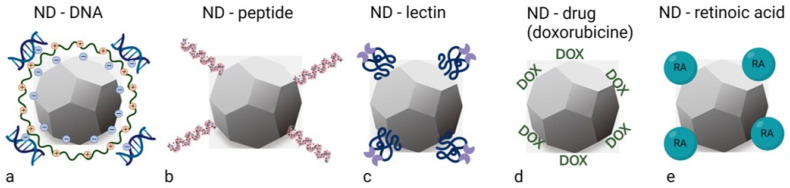
Useful functionalisation of NDs by non-covalent binding of DNA (**a**), peptide (**b**), lectin (**c**), drugs such as doxorubicin (DOX; (**d**)) and retinoic acid (RA; (**e**)) [6,21,48]. The illustration was created by the authors using the BioRender app.

Most approaches to ND surface modification have been developed for DNDs, as synthesis method for DNDs was developed early on and DNDs are widely available commercially [6]. Although there are differences between the surface components of DNDs and FNDs, the homogenisation and further chemical reactions developed for DNDs can generally also be applied to the surface of FNDs [32]. Notwithstanding the differences in synthesis and NV formation between DNDs and HPHT nanodiamonds, the discrepancy between them is slowly disappearing as synthesis and purification methods continue to evolve. The initial surface structure of ND depends on the synthesis and purification methods. Thereafter, the main methods that allow the heterogeneous ND surface to be reconstructed to obtain a chemically homogeneous surface with desired functional groups are hydroxylation and carboxylation (Figure 1a,b) [21]. These two approaches lead to hydroxylated and carboxylated NDs that are hydrophilic and have high dispersibility in aqueous solution, with typical zeta potentials of −30 to −50 mV and about +40 mV, respectively [21]. A recent publication on negatively charged NDs has shown that, compared to positively charged NDs of the same size, they are more resistant to aggregation and exhibit high colloidal stability in a wide range of pH values and salt concentrations [33]. Negatively charged NDs have a more homogeneous surface and their functional groups are mostly carboxy units. In contrast, positively charged NDs have an inhomogeneous surface that contains a mixture of different functional groups. This is important for further functionalisation steps with biomolecules such as bovine serum albumin (BSA), where surface differences are to be hidden [33]. However, hydroxylation and carboxylation of NDs are insufficient to suppress aggregation in various cell culture media and exhibit non-specific protein adsorption. To avoid these problems, coatings are applied to the NDs by using the carboxy, hydroxy and amino groups as scaffolds for the reaction (Figure 1). The potential biomedical applications of the resulting material can be realised by one of two coating methods: covalent or non-covalent binding of the bioactive structure on the surface of ND. Covalent binding is usually achieved by conventional organic chemistry. Non-covalent binding, on the other hand, is a rather simple and flexible approach to modify the surface of ND with the desired functional molecules by physical adsorption. The most common covalent functionalisation is by silica and polymers such as polyethylene glycol or polyglycerol, while non-covalent binding includes various functional materials such as DNA, siRNA, peptides, proteins, lipids, fluorochromes, drugs (such as doxorubicin) and retinoic acid (Figure 2) [34,35,36,37,38,39,40,41]. In particular, various glycoproteins are commonly used, e.g., lectins that target specific sugar residues [42,43]. Since cancer transformation is associated with changes in sugar residues on the cell surface, lectins have recently been proposed as tools for targeted drug delivery [44,45,46,47].

## 3. Prospective of Nanodiamonds in Bladder Cancer Management

Bladder cancer is among most common cancers worldwide, with nearly 600,000 new cases in 2020 [49,50]. It is a heterogeneous disease, usually originating from the urothelium, the inner lining of the bladder wall, and presenting a wide spectrum of pathologies and clinical outcomes [51]. For therapeutic purposes, bladder cancer is divided into non-muscle-invasive and muscle-invasive cancers, depending on how deeply it has invaded the bladder wall [52]. Non-muscle-invasive bladder cancer is usually treated by transurethral resection of the bladder (TURB). Sometimes TURB is followed by intravesical instillation of chemotherapeutic agents (e.g., mitomycin C) or Bacillus Calmette Guerin (BCG) [53]. Non-muscle-invasive bladder cancer is characterised by an extremely high recurrence rate (50–70%), requiring systematic follow-up of patients decades after initial treatment [54]. Bladder cancer treatment is the most expensive of all cancers due to the need for invasive long term follow up of the patients, while research into innovative diagnostic, prevention and treatment strategies is underfunded. The result is the lack of high-quality studies conducted with NDs in bladder cancer cells in vitro and in vivo.

In general, the treatment and diagnosis of all types of cancer is currently one of the most pressing problems in medicine. The targeted administration of drugs that directly target cancer cells significantly reduces the risk of harming normal cells of the neighbouring tissue, thus reducing the side effects of the drug [55,56]. A modern solution for targeted delivery is the use of monoclonal antibodies, which recognise specific antigens on the surface of cancer cells to achieve the intended therapeutic effect [57]. A promising example of such targeted delivery is also the use of lectins that selectively bind to specific sugar residues of cancer cells [45,58]. Currently, NDs play an important role in the search for the most effective carriers for different types of drugs. Research into ND carriers aims to develop innovative drug delivery systems that will ensure high efficacy with minimal side effects. The most important criteria here are the timing of administration, the dosage and the release pattern of the drug [59,60]. Advanced drug delivery systems exhibit improved bioavailability by preventing premature degradation, improving drug absorption, maintaining drug concentration within the therapeutic window by controlling the rate of drug release and minimalizing side effects through targeted delivery [59]. On the other hand, a recent review analysing data published over the last 10 years concluded that only 0.7% (median) of the administered nanoparticle dose reaches a solid tumour [61]. In the case of bladder cancer, intravesical therapy is a specific mode of administration that uses a urinary catheter to deliver chemotherapy directly into the bladder cavity, avoiding systemic administration [62]. However, intravesical chemotherapy is limited by the short retention time between micturition phases [62].

Other mechanisms for the delivery and accumulation of nanoparticles in the tumour are also known. For example, passive tumour targeting, co-called the EPR effect (enhanced permeability and retention) [63]. The EPR effect can be used in later stages of tumour development as it depends on a well-developed tumour vasculature. Recently, however, it has been demonstrated that NDs can induce higher vascular permeability independently of tumour-induced EPR [64]. Interestingly, among NDs with different surfaces, aminated NDs elicited the highest degree of vascular permeability compared to carboxylated NDs and NDs without surface modifications. The proposed mechanism involves a ND-triggered cascade of cellular processes leading to the opening of tight junctions between endothelial cells. This effect is reversible and the tight junctions return to normal when the ND treatment ends [65].

Once near the surface of the cancer cells, NDs are internalised by endocytosis [5,55]. Endocytotic processes are highly enhanced in urothelial cancer cells while largely depressed in normal urothelial cells and so offer an additional way to selectively target cancer cells [66]. Cancer cells in general internalise large amounts of extracellular fluid by receptor-mediated macropinocytosis, or they may use receptor-mediated clathrin or caveolin-mediated endocytosis [67]. Therefore, NDs coated with different ligands could enhance delivery into a cancer cell, with minimal effects on normal cells [68]. One of the ways to induce cancer cells to internalise larger amounts of drugs than normal cells is to target changes of sugar residues of membrane glycoproteins [44]. This fact could be used as a characteristic biomarker of bladder carcinogenesis as well as for targeted drug delivery [69,70,71]. Despite the recognised potential of lectin for targeted drug delivery [58,72,73,74], lectins have not yet been adsorbed to NDs.

**Figure 3:**
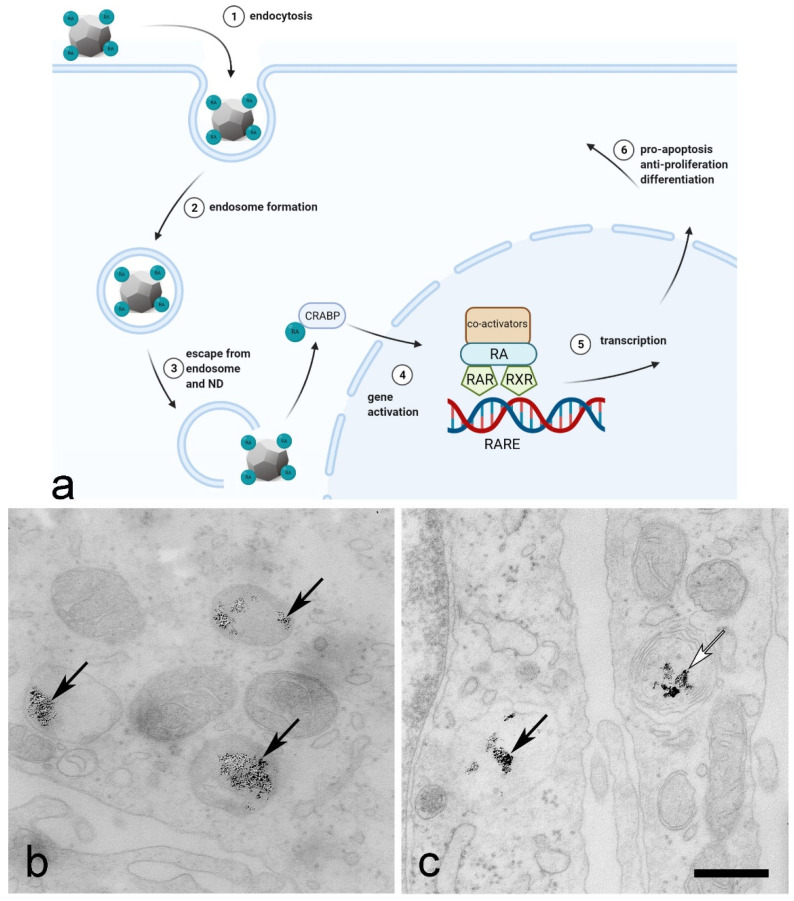
Endocytosis and intracellular pathway of retinoic acid (RA) conjugated to ND. (**a**) After endosome formation in the cell, the crucial step is the release of RA from the endosome and its binding to the cellular retinoic acid-binding protein (CRABP). CRABP transports RA into the nucleus, where it can form large protein complexes consisting of co-activators and the retinoic acid receptor (RAR) and retinoid X receptor (RXR), activating RA response elements (RARE). (**b**,**c**) Internalisation assay performed with two human cell lines: non-invasive papillary urothelial cancer cells RT4 (**b**) and invasive urothelial cancer cells T24 (**c**). For both cell lines, DNDs were added to the culture medium at a concentration of 11 µg/mL for 24 h and cells were prepared for transmission electron microscopy. DNDs were observed in endocytotic compartments (black arrows; (**b**,**c**)) and in multilamellar bodies (white arrow; **c**). A detailed analysis of the endocytosis of DNDs is published in [5]. Scale bars: 500 nm. The illustration was created by the authors using the BioRender app and our own TEM images.

Since retinoids have the potential to reduce the incidence and recurrence of bladder cancer and improve therapy [75], the transport of retinoic acid bound to NDs into cancer urothelial cells is a promising mechanism for the treatment of bladder cancer (Figure 3a). Once inside the cell, retinoids adsorbed to NDs cannot simply be expelled by cell exocytosis or other cell efflux mechanisms. Instead, they are slowly desorbed from the NDs within the endocytotic compartment, maintaining therapeutic concentrations. This effect could be exploited in the case of cancer cell resistance to conventional chemotherapeutic agents [76]. Indeed, therapeutic efflux is the most common mechanism of cancer cell chemoresistance, limiting the efficacy of cytotoxic drugs. Endosomal release is caused by low pH in the endosomal system, which promotes desorption of the drug from ND [77] and escape from the endosome to reach the target compartment. Retinoic acid, for example, binds to the cellular retinoic acid-binding protein (CRABP) and is subsequently transported to the nucleus. In the cell nucleus, RA regulates gene expression by binding to retinoic acid receptors (RARs) and retinoid X receptors (RXRs) (Figure 3a), which in turn activate the transcription of their downstream target genes (Figure 3a). The different isomers of RA activate different receptors, leading to different biological effects such as pro-apoptosis, anti-proliferation and differentiation of cells [75,78,79].

One of the most commonly used and studied chemotherapeutic agents is doxorubicin (DOX), an apoptosis-inducing drug that can be conjugated to the surface of NDs. Such ND-DOX has been applied to numerous cancer cells such as colorectal carcinoma cells, tetracycline-resistant leukaemia cells, brain tumour cells, liver carcinoma, breast adenocarcinoma and also in vivo mouse models of liver and breast cancer [76,77,80,81,82,83,84]. The adsorption of DOX to the NDs and its reversible release were achieved by regulating the Cl^-^ ion concentration, and the NDs were found to be able to efficiently transport the drug into living cells [84]. The apoptosis-inducing mechanisms driven by the NDs functionalised with DOX were preserved and the effect of DOX was reduced by ND-mediated DOX sequestration. This result proves that DOX functionality is preserved, but the result does not show that the ND-DOX composite is superior to the pure drug at the same dosage [84,85].

On the other hand, the pH also influences the release of the DOX from the NDs. In an alkaline environment (pH = 8), the carboxyl group of ND together with the amino group of DOX form a stable and non-covalent bond on the surface of ND, which served as a simple physical adsorption. In an acidic environment of an endocytic compartment such as the early or late endosome, the NDs carboxyl group is ionised, effectively releasing DOX. Thereafter, DOX is affirmatively absorbed into the cytoplasm and later into the nucleus [83]. Interestingly, a pH-sensitive nanocomplex has been developed for the co-administration of DOX and all-trans retinoic acid (ATRA)-based NDs (DOX-ATRA-NDs) to improve intracellular retention of the drugs. Ultrasound has been also used to enhance the vascular penetration of DOX-ATRA-NDs in liver and breast tumours of a mouse model [86]. The results of the study showed that the combined therapy caused greater apoptosis of cancer cells and inhibition of tumour growth compared to unmodified chemotherapeutic agents in both tumour models. DOX-ATRA-NDs have been therefore considered by the authors to have great potential for clinical applications [86]. With regard to bladder cancer, a recent study investigated chitosan-coated DOX-loaded NDs as a mucoadhesive platform for intravesical delivery of DOX [41]. The effects of chitosan molecular weight and pH on the particle size and surface charge of these NDs were studied, and it was found that the drug loading was over 90%, the particles had a size of less than 150 nm and their colloidal stability was good. In addition, good acid-favoured drug release was observed, but the stability of the NDs in cell culture media was limited. Therefore, stabilisation of the NDs with tripolyphosphate was performed, resulting in a stronger cytotoxic effect and higher drug retention in bovine bladder ex vivo compared to free DOX and uncoated NDs [41]. All in all, ND-DOX is a promising system for mucosal delivery of anticancer drugs by intravesical administration. On the other hand, intraperitoneally injected NDs penetrated the blood–brain barrier in rats and could be used for brain tumours [65,87]. However, only a small proportion of injected NDs were observed in rat brain, while a larger proportion were found in liver, spleen and lymph nodes [87]. Future research is needed to assess the long-term effects of NDs on the different organ systems.

The search for newer, innovative and more effective treatments could lead to NDs being used in the treatment of bladder cancer in the future. In addition, NDs could also be used to treat bacterial cystitis due to their antibacterial and anti-inflammatory properties.

## 4. Nanodiamond Platforms for the Treatment of Bacterial Cystitis

Urinary tract infections (UTI) are the second most common infection in women, causing over 8 million UTI cases annually in the United States [88]. The causative agent in over 70% of UTI cases is uropathogenic *Escherichia coli* (UPEC). Patients with UTI are most often prescribed antibiotic treatment. Despite antibiotic treatment, the probability of a female patient having a second UTI within six months is 25%. It was found that in most cases the initial infection and recurrence are caused by the genetically identical strain of UPEC [89]. This suggest that the intracellular bacterial reservoirs within the urothelial cells are involved and that recurrent episodes originate from these reservoirs.

**Figure 4:**
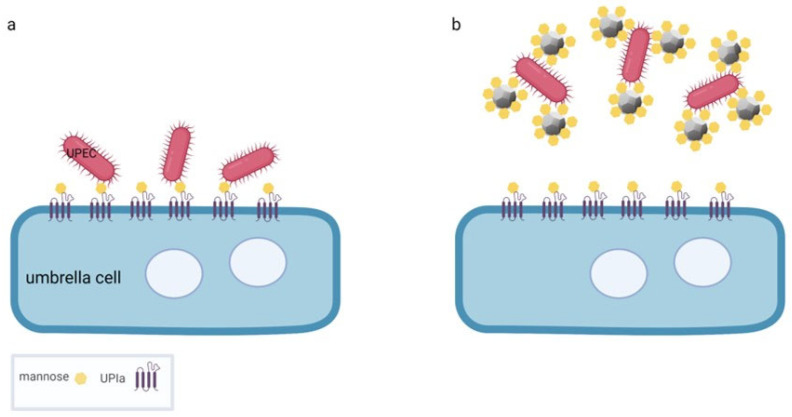
Possible mechanism to prevent UPEC from attaching to urothelial cells. (**a**) The apical plasma membrane of the umbrella cell has transmembrane proteins uroplakin Ia (UPIa) with four transmembrane domains and mannose units to which UPEC attach before entering the cell. (**b**) NDs conjugated with mannose units could bind to UPEC, occupying the binding sites and thus preventing their attachment to umbrella cells. The illustration was created by the authors using the BioRender app.

**Figure 5:**
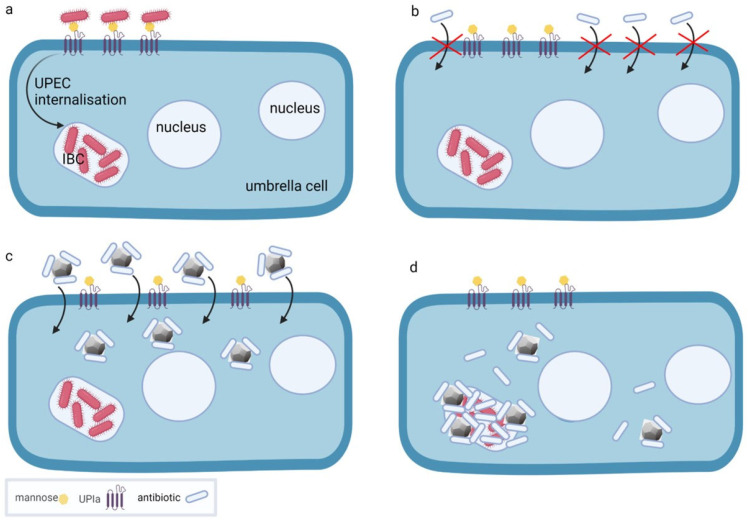
Possible mechanism for elimination of the intracellular bacterial community (IBC) of UPEC in superficial urothelial umbrella cells. (**a**) The apical plasma membrane of the umbrella cell has transmembrane proteins uroplakin Ia (UPIa) with four transmembrane domains and mannose units to which UPEC attach before entering the cell and forming IBC within the cell. (**b**) Antibiotics cannot penetrate the apical plasma membrane of the umbrella cell and therefore cannot reach and eliminate UPEC within the IBC. (**c**) Antibiotics bound to NDs can penetrate the apical plasma membrane of the umbrella cell. (**d**) Once inside the cell, the antibiotics reach the IBC, dissociate from the NDs and eliminate the intracellular reservoir of UPEC within the IBC. The illustration was created by the authors using the BioRender app.

UPEC penetrate superficial, highly differentiated, so-called umbrella cells and the underlying less differentiated intermediate and basal urothelial cells by two different mechanisms. The umbrella cells are infected by direct penetration of UPEC through the apical plasma membrane. This membrane is highly specialised and organised into urothelial plaques composed of four major transmembrane proteins, the uroplakins. Uroplakin Ia presents a high level of terminally exposed mannose residues, which serve as the attachment point for UPEC adhesion (Figure 4) [90,91,92]. This adhesion event allows host and pathogen recognition by specific proteins called adhesins. In UPEC, the best known adhesin is FimH. FimH is located at the tip of type 1 fimbriae, filament-like structures up to two μm in length that are distributed over the surface of the bacterium [93]. After attachment, the bacteria penetrate the apical plasma membrane of the umbrella cells and begin to multiply rapidly in the umbrella cells. Soon, a biofilm-like accumulation known as an intracellular bacterial community (IBC) forms (Figure 5a) [94]. The development of IBCs may enhance the ability of UPEC to successfully survive within cells by shielding them from the host immune system [95]. The occasional exit of UPEC from the umbrella cells allows UPEC to invade the new, uninfected urothelial cells at all different stages of differentiation. The second mechanism of UPEC internalisation occurs in less differentiated urothelial cells of the intermediate and basal cell layers. After being phagocytosed, UPEC usually enter a quiescent state [94]. The dormant nature and intracellular localisation of these bacteria protects them from most antibiotic treatments (Figure 5b) [95]. These dormant intracellular UPEC reservoirs can persist for months without clinical symptoms and are undetectable in urine [95]. Differentiation of less differentiated urothelial cells harbouring dormant bacteria can trigger the regrowth of UPEC, leading to the development and spread of IBCs in the umbrella cells and the recurrence of clinical symptoms [96]. The ineffectiveness of antibiotics to completely eliminate UPEC from urothelial tissue, and thus the increasing threat of bacterial resistance to antibiotics, necessitates therapeutic strategies that support antibiotic treatment of both active and dormant stages of UTI.

Since interaction between pathogenic bacteria and host cells is the critical step prior to host colonisation, prevention of such contact has been used as a promising strategy to treat bacterial bladder infections. Inhibition of FimH-mediated adhesion to umbrella cells is generally used as an effective antibiotic alternative or supportive strategy to reduce UPEC-related infections. Unfortunately, monovalent mannose suspensions have very little inhibitory effect in preventing FimH-mediated bacterial adhesion to urothelial cells [97]. ND particles functionalised with mannose moieties have been shown to efficiently inhibit UPEC type 1 fimbriae-mediated adhesion to urothelial cells (Figure 4). Of additional value, these mannose-modified NDs reduce UPEC biofilm formation, which has not been observed with other multivalent or monovalent mannose glycans [97]. Indeed, NDs covalently modified with mannose moieties have been successfully tested to affect UPEC colonies in invasive urothelial cancer T24 cells [98].

In addition to using NDs as carriers for glycan groups, they have been successfully used as attachment particles that are bacteriostatic or bactericidal for both extracellular and intracellular UPEC colonies [99]. In this study, an invasive strain of UPEC causing recurrent UTI and acid-purified 6 nm DNDs were used. DNDs effectively eliminated UPEC from T24 cells, which were used as an in vitro model for less differentiated urothelial cells. DNDs reduced the number of UPEC both extracellularly and of those internalised in the cells in a dose-dependent manner. The DNDs were internalised within two hours by an actin-dependent mechanism and this internalisation reduced the number of intracellular UPEC in T24 cells. The authors suggested that the physical properties of the DND surface, particularly its positive charge, are responsible for the bactericidal efficacy [99]. These results suggest that 6 nm DNDs are promising candidates for the treatment of recurrent UTI, as shown schematically in Figure 5.

## 5. Diagnostics, Therapy and Theranostics Using Nanodiamonds in Diseases of the Urinary Bladder

All diseases of the urinary bladder share the extreme difficulty of reaching the affected cells with conventional treatment methods, both in the case of cancer transformation and bacterial infections. The main obstacle is the exceptionally impermeable apical plasma membrane of the umbrella cells, which prevents the penetration of cytostatics to cancer cells nested in lower layers of urothelium [100] and antibiotics [95]. Surprisingly, the NDs have been shown to be able to penetrate the urothelial cells [5], possibly due to their positive surface charge, and thus can be used to treat various bladder diseases, including infections caused by intracellular colonies of UPEC. Furthermore, as the NDs are capable of labelling both cancer cells and infected cells, they can be used to label cells of interest as well as to treat common bladder diseases and can thus be used as theranostics. Among the different types of NDs, DNDs and FNDs have the greatest potential to be used both as markers of cells in focus and carriers of specific drugs that can be targeted in hard-to-reach tissues.

Detection and specific labelling of cancer cells in the bladder is an important step prior to transurethral resection of tumours, allowing surgeons to efficiently remove the cancerous tissue. Although white light cystoscopy remains the diagnostic standard for the detection of bladder tumours, flat cancerous lesions can often be missed [101]. For this reason, new techniques have been developed to increase the sensitivity for detecting urothelial cancer cells. Currently, the most successfully used method is photodynamic diagnosis. This method has a significantly higher sensitivity for the detection of urothelial cancer cells than white light cystoscopy (92% vs. 71%) [102]. However, photodynamic diagnosis has a relatively high rate of false positives [103]. To avoid this problem, it was proposed to investigate another highly specific property difference between normal and cancer urothelial cells, namely the ability to internalise the nanoparticles from the apical surface. Due to the highly pronounced difference in the ability to internalise nanoparticles between normal and cancer cells in urothelial tissue [66], the entire population of cancer cells can be specifically labelled by using FNDs and easily detected by fluorescence cystoscopy. Additionally, attachment of cytostatics to FNDs might in this way specifically affect only cancer cells and act as theranostics.

To the FNDs with a functionalised surface [6], various glycan groups or/and antibiotics can be added. By attaching to FimH, FNDs both label the bacteria and reduce their attachment to the urothelial cells and to each other [104]. Such prevention of extracellular and intracellular biofilm formation makes UPEC much more susceptible to antibiotics. At the same time, the bacteria can be visualised by fluorescence cystoscopy and the success of antibiotic treatment can be assessed in this way.

Lien et al. suggested that fluorescent and magnetic NDs (FMNDs) could be used to label and track bladder cancer cells [105]. In their study, FMNDs were in fact used on lung cancer cells (A549). The FMND-containing lung cancer cells were separated based on their fluorescent and magnetic properties using a flow cytometer and a magnetic rack, respectively. The results of the study showed that the cell morphology, viability, growth ability and total protein expression profiles in the FMND-containing cells were similar to those of the original cells [105]. In the study by Torelli et al., conjugates of 140 nm FNDs and a single-chain version of vascular endothelial growth factor (VEGF) were used to target VEGF receptors in mammary carcinoma tumours in vitro and in vivo (induced 4T1 carcinoma in Balb/c mice) [40]. In cell cultures, the FND-scVEGF conjugates retained high affinity for VEGF receptors. In experiments with mice, preferential accumulation of FND-scVEGF in tumours was observed compared to accumulation of untargeted FNDs [40]. FNDs were determined in tissue by microspectroscopy via the unique spectral shape of NV induced fluorescence [40]. Based on their findings, the authors propose the use of targeted FNDs for diagnostic imaging [40], which could also be used for diseases of the bladder.

## 6. Comparison of Nanodiamonds with Other Nanoparticles

In recent decades, various types of nanoparticles (NPs) have been developed as contrast agents for cell tracking in biomedical examinations, in addition to NDs, as described by Jen-Shyang Ni et al. in 2020 [106]. The most popular NPs include superparamagnetic iron oxide NPs (SPIONs), silver NPs and quantum dots (QDs). These NPs are particularly interesting because they are used for long-term cell tracking of biomedical processes. Some of them have already undergone clinical trials using SPIONs for magnetic particle imaging (MPI) of pancreatic cancer and silver NPs for catheter-related infections in central venous catheters (information from https://clinicaltrials.gov). Quantum dots are also becoming increasingly popular for fluorescent labelling of cells and as labels for antibodies. In particular, the inorganic QDs are known for their long fluorescence lifetime, high fluorescence quantum yield and photostability [107]. However, due to the toxicity of the classical QDs, new QDs have been developed, such as silver sulphide QDs with lower toxicity, but excellent photostability [108]. Compared to the aforementioned NPs, fluorescent nanodiamonds have better spatial resolution, allowing organelle tracking, and very low toxicity, allowing long-term cell tracking. As found in terminally differentiated urothelial cells, NDs are so far the only NP that can penetrate the apical plasma membrane [5] and thus could be used for bacterial tracking and elimination.

## 7. Conclusions

The treatment of bladder cancer and bacterial cystitis has not improved significantly in the last decade, although the social and economic burden of both diseases is high, especially in developed countries. In view of this problem, the search for innovative approaches is crucial, and NDs are one of the promising tools. The great biocompatibility, low toxicity and wide range of possible specific functionalisation of NDs are their greatest advantages. In addition, DNDs and FNDs could also be used for theranostics, as they allow visualisation of cancer urothelial cells as well as UPEC-infected urothelial cells, and also show potential for the treatment of bladder cancer and bacterial cystitis. However, in vivo studies show that NDs accumulate in animal organs and there are no confirmed studies on the long-term effects of their release from organs. This is a real problem, especially as many patients require repeated therapies. Intravesical application of therapeutic NDs can be used for bladder cancer and bacterial cystitis. Such application allows direct contact of the drug conjugated to the NDs with the diseased urothelium and immediate excretion of the NDs with micturition. Therefore, in the case of the bladder, there is no concern about accumulation of NDs in other organs of the body. However, in our opinion, the main problem is the small number of studies using NDs to treat bladder cancer and bacterial cystitis. In addition, standardised ND materials and drug delivery approaches are needed to enable clinical-level applications.

## References

[1] Volkov K.V., Danilenko V.V., Elin V.I. (1990). Synthesis of diamond from the carbon in the detonation products of explosives. Combust. Explos. Shock. Waves.

[2] Holt K.B. (2007). Diamond at the nanoscale: Applications of diamond nanoparticles from cellular biomarkers to quantum computing. Philos. Trans. A Math. Phys. Eng. Sci..

[3] Danilenko V.V. (2004). On the history of the discovery of nanodiamond synthesis. Phys. Solid State.

[4] Yu S.J., Kang M.W., Chang H.C., Chen K.M., Yu Y.C. (2005). Bright fluorescent nanodiamonds: No photobleaching and low cytotoxicity. J. Am. Chem. Soc..

[5] Zupančič D., Kreft M.E., Grdadolnik M., Mitev D., Iglič A., Veranič P. (2018). Detonation nanodiamonds are promising nontoxic delivery system for urothelial cells. Protoplasma.

[6] Jung H.S., Neuman K.C. (2021). Surface Modification of Fluorescent Nanodiamonds for Biological Applications. Nanomaterials.

[7] Ashfold M.N.R., Goss J.P., Green B.L., May P.W., Newton M.E., Peaker C.V. (2020). Nitrogen in Diamond. Chem. Rev..

[8] Vaijayanthimala V., Lee D.K., Kim S.V., Yen A., Tsai N., Ho D., Chang H.C., Shenderova O. (2015). Nanodiamond-mediated drug delivery and imaging: Challenges and opportunities. Expert Opin. Drug Deliv..

[9] Chipaux M., van der Laan K.J., Hemelaar S.R., Hasani M., Zheng T., Schirhagl R. (2018). Nanodiamonds and Their Applications in Cells. Small.

[10] Vlasov I.I., Shiryaev A.A., Rendler T., Steinert S., Lee S.Y., Antonov D., Voros M., Jelezko F., Fisenko A.V., Semjonova L.F. (2014). Molecular-sized fluorescent nanodiamonds. Nat. Nanotechnol..

[11] Havlik J., Petrakova V., Rehor I., Petrak V., Gulka M., Stursa J., Kucka J., Ralis J., Rendler T., Lee S.-Y. (2013). Boosting nanodiamond fluorescence: Towards development of brighter probes. Nanoscale.

[12] Boudou J.P., Curmi P.A., Jelezko F., Wrachtrup J., Aubert P., Sennour M., Balasubramanian G., Reuter R., Thorel A., Gaffet E. (2009). High yield fabrication of fluorescent nanodiamonds. Nanotechnology.

[13] Chang Y.R., Lee H.Y., Chen K., Chang C.C., Tsai D.S., Fu C.C., Lim T.S., Tzeng Y.K., Fang C.Y., Han C.C. (2008). Mass production and dynamic imaging of fluorescent nanodiamonds. Nat. Nanotechnol..

[14] Fu C.C., Lee H.Y., Chen K., Lim T.S., Wu H.Y., Lin P.K., Wei P.K., Tsao P.H., Chang H.C., Fann W. (2007). Characterization and application of single fluorescent nanodiamonds as cellular biomarkers. Proc. Natl. Acad. Sci. USA.

[15] Shenderova O.A., Shames A.I., Nunn N.A., Torelli M.D., Vlasov I., Zaitsev A. (2019). Review Article: Synthesis, properties, and applications of fluorescent diamond particles. J. Vac. Sci. Technol. B Nanotechnol. Microelectron..

[16] Davies G., Lawson S.C., Collins A.T., Mainwood A., Sharp S.J. (1992). Vacancy-related centers in diamond. Phys. Rev. B Condens Matter.

[17] Fang C.Y., Chang C.C., Mou C.Y., Chang H.C. (2015). Preparation and Characterization of Ion-Irradiated Nanodiamonds as Photoacoustic Contrast Agents. J. Nanosci. Nanotechnol..

[18] Tzeng Y.K., Zhang J.L., Lu H., Ishiwata H., Dahl J., Carlson R.M., Yan H., Schreiner P.R., Vuckovic J., Shen Z.X. (2017). Vertical-Substrate MPCVD Epitaxial Nanodiamond Growth. Nano Lett..

[19] Dahl J.E., Moldowan J.M., Wei Z., Lipton P.A., Denisevich P., Gat R., Liu S., Schreiner P.R., Carlson R.M. (2010). Synthesis of higher diamondoids and implications for their formation in petroleum. Angew. Chem. Int. Ed. Engl..

[20] Osswald S., Yushin G., Mochalin V., Kucheyev S.O., Gogotsi Y. (2006). Control of sp2/sp3 carbon ratio and surface chemistry of nanodiamond powders by selective oxidation in air. J. Am. Chem. Soc..

[21] Krueger A., Lang D. (2012). Functionality is Key: Recent Progress in the Surface Modification of Nanodiamond. Adv. Funct. Mater..

[22] Kruger A. (2006). Hard and soft: Biofunctionalized diamond. Angew. Chem. Int. Ed. Engl..

[23] Jiang T., Xu K. (1995). FTIR study of ultradispersed diamond powder synthesized by explosive detonation. Carbon.

[24] Krüger A., Kataoka F., Ozawa M., Fujino T., Suzuki Y., Aleksenskii A.E., Vul’ A.Y., Ōsawa E. (2005). Unusually tight aggregation in detonation nanodiamond: Identification and disintegration. Carbon.

[25] Krüger A., Liang Y., Jarre G., Stegk J. (2006). Surface functionalisation of detonation diamond suitable for biological applications. J. Mater. Chem..

[26] Hens S.C., Cunningham G., Tyler T., Moseenkov S., Kuznetsov V., Shenderova O. (2008). Nanodiamond bioconjugate probes and their collection by electrophoresis. Diam. Relat. Mater..

[27] Martín R., Heydorn P.C., Alvaro M., Garcia H. (2009). General Strategy for High-Density Covalent Functionalization of Diamond Nanoparticles Using Fenton Chemistry. Chem. Mater..

[28] Girard H.A., Petit T., Perruchas S., Gacoin T., Gesset C., Arnault J.C., Bergonzo P. (2011). Surface properties of hydrogenated nanodiamonds: A chemical investigation. Phys. Chem. Chem. Phys..

[29] Ando T., Ishii M., Kamo M., Sato Y. (1993). Thermal hydrogenation of diamond surfaces studied by diffuse reflectance Fourier-transform infrared, temperature-programmed desorption and laser Raman spectroscopy. J. Chem. Soc. Faraday Trans..

[30] Arnault J.C., Girard H.A. (2017). Hydrogenated nanodiamonds: Synthesis and surface properties. Curr. Opin. Solid State Mater. Sci..

[31] Sotowa K.-I., Amamoto T., Sobana A., Kusakabe K., Imato T. (2004). Effect of treatment temperature on the amination of chlorinated diamond. Diam Relat Mater.

[32] Wolcott A., Schiros T., Trusheim M.E., Chen E.H., Nordlund D., Diaz R.E., Gaathon O., Englund D., Owen J.S. (2014). Surface Structure of Aerobically Oxidized Diamond Nanocrystals. J. Phys. Chem. C Nanomater. Interfaces.

[33] Bradac C., Rastogi I.D., Cordina N.M., Garcia-Bennett A., Brown L.J. (2018). Influence of surface composition on the colloidal stability of ultra-small detonation nanodiamonds in biological media. Diam. Relat. Mater..

[34] Guerrero-Martínez A., Pérez-Juste J., Liz-Marzán L.M. (2010). Recent Progress on Silica Coating of Nanoparticles and Related Nanomaterials. Adv. Mater..

[35] Zhang X., Fu C., Feng L., Ji Y., Tao L., Huang Q., Li S., Wei Y. (2012). PEGylation and polyPEGylation of nanodiamond. Polymer.

[36] Shi Y., Liu M., Wang K., Huang H., Wan Q., Tao L., Fu L., Zhang X., Wei Y. (2015). Direct surface PEGylation of nanodiamond via RAFT polymerization. Appl. Surf. Sci..

[37] Neburkova J., Vavra J., Cigler P. (2017). Coating nanodiamonds with biocompatible shells for applications in biology and medicine. Curr. Opin. Solid State Mater. Sci..

[38] Zhang X.-Q., Chen M., Lam R., Xu X., Osawa E., Ho D. (2009). Polymer-Functionalized Nanodiamond Platforms as Vehicles for Gene Delivery. ACS Nano.

[39] Alhaddad A., Adam M.-P., Botsoa J., Dantelle G., Perruchas S., Gacoin T., Mansuy C., Lavielle S., Malvy C., Treussart F. (2011). Nanodiamond as a Vector for siRNA Delivery to Ewing Sarcoma Cells. Small.

[40] Torelli M.D., Rickard A.G., Backer M.V., Filonov D.S., Nunn N.A., Kinev A.V., Backer J.M., Palmer G.M., Shenderova O.A. (2019). Targeting Fluorescent Nanodiamonds to Vascular Endothelial Growth Factor Receptors in Tumor. Bioconjug. Chem..

[41] Ali M.S., Metwally A.A., Fahmy R.H., Osman R. (2020). Chitosan-coated nanodiamonds: Mucoadhesive platform for intravesical delivery of doxorubicin. Carbohydr. Polym..

[42] Sharon N., Lis H. (1972). Lectins: Cell-agglutinating and sugar-specific proteins. Science.

[43] Sharon N., Lis H. (2004). History of lectins: From hemagglutinins to biological recognition molecules. Glycobiology.

[44] Visnjar T., Romih R., Zupancic D. (2019). Lectins as possible tools for improved urinary bladder cancer management. Glycobiology.

[45] Zupancic D., Kreft M.E., Romih R. (2014). Selective binding of lectins to normal and neoplastic urothelium in rat and mouse bladder carcinogenesis models. Protoplasma.

[46] Neutsch L., Plattner V.E., Polster-Wildhofen S., Zidar A., Chott A., Borchard G., Zechner O., Gabor F., Wirth M. (2011). Lectin mediated biorecognition as a novel strategy for targeted delivery to bladder cancer. J. Urol..

[47] Plattner V.E., Wagner M., Ratzinger G., Gabor F., Wirth M. (2008). Targeted drug delivery: Binding and uptake of plant lectins using human 5637 bladder cancer cells. Eur. J. Pharm. Biopharm..

[48] Terada D., Genjo T., Segawa T.F., Igarashi R., Shirakawa M. (2020). Nanodiamonds for bioapplications-specific targeting strategies. Biochim. Biophys. Acta Gen. Subj..

[49] Richters A., Aben K.K.H., Kiemeney L. (2020). The global burden of urinary bladder cancer: An update. World J. Urol..

[50] Global Cancer Observatory (International Agency for Research on Cancer WHO) (2020). Cancer Fact Sheets (Bladder). https://gco.iarc.fr/.

[51] Magers M.J., Lopez-Beltran A., Montironi R., Williamson S.R., Kaimakliotis H.Z., Cheng L. (2019). Staging of bladder cancer. Histopathology.

[52] Kamat A.M., Hahn N.M., Efstathiou J.A., Lerner S.P., Malmstrom P.U., Choi W., Guo C.C., Lotan Y., Kassouf W. (2016). Bladder cancer. Lancet.

[53] Babjuk M., Burger M., Compérat E.M., Gontero P., Mostafid A.H., Palou J., van Rhijn B.W.G., Rouprêt M., Shariat S.F., Sylvester R. (2019). European Association of Urology Guidelines on Non-muscle-invasive Bladder Cancer (TaT1 and Carcinoma In Situ)—2019 Update. Eur. Urol..

[54] Ritch C.R., Velasquez M.C., Kwon D., Becerra M.F., Soodana-Prakash N., Atluri V.S., Almengo K., Alameddine M., Kineish O., Kava B.R. (2020). Use and Validation of the AUA/SUO Risk Grouping for Nonmuscle Invasive Bladder Cancer in a Contemporary Cohort. J. Urol..

[55] Lisik K., Krokosz A. (2021). Application of Carbon Nanoparticles in Oncology and Regenerative Medicine. Int. J. Mol. Sci..

[56] Ji Z., Lin G., Lu Q., Meng L., Shen X., Dong L., Fu C., Zhang X. (2012). Targeted therapy of SMMC-7721 liver cancer in vitro and in vivo with carbon nanotubes based drug delivery system. J. Colloid Interface Sci..

[57] Xu S., Cui F., Huang D., Zhang D., Zhu A., Sun X., Cao Y., Ding S., Wang Y., Gao E. (2019). PD-L1 monoclonal antibody-conjugated nanoparticles enhance drug delivery level and chemotherapy efficacy in gastric cancer cells. Int. J. Nanomed..

[58] Neutsch L., Eggenreich B., Herwig E., Marchetti-Deschmann M., Allmaier G., Gabor F., Wirth M. (2014). Biomimetic delivery strategies at the urothelium: Targeted cytoinvasion in bladder cancer cells via lectin bioconjugates. Pharm. Res..

[59] Zhang Y., Chan H.F., Leong K.W. (2013). Advanced materials and processing for drug delivery: The past and the future. Adv. Drug Deliv. Rev..

[60] Chow E.K., Ho D. (2013). Cancer nanomedicine: From drug delivery to imaging. Sci. Transl. Med..

[61] Wilhelm S., Tavares A.J., Dai Q., Ohta S., Audet J., Dvorak H.F., Chan W.C.W. (2016). Analysis of nanoparticle delivery to tumours. Nat. Rev. Mater..

[62] Patel S.G., Cohen A., Weiner A.B., Steinberg G.D. (2015). Intravesical therapy for bladder cancer. Expert Opin. Pharm..

[63] Seymour L.W. (1992). Passive tumor targeting of soluble macromolecules and drug conjugates. Crit. Rev. Ther. Drug Carr. Syst..

[64] Setyawati M.I., Mochalin V.N., Leong D.T. (2016). Tuning Endothelial Permeability with Functionalized Nanodiamonds. ACS Nano.

[65] Turcheniuk K., Mochalin V.N. (2017). Biomedical applications of nanodiamond (Review). Nanotechnology.

[66] Lojk J., Bregar V.B., Strojan K., Hudoklin S., Veranic P., Pavlin M., Kreft M.E. (2018). Increased endocytosis of magnetic nanoparticles into cancerous urothelial cells versus normal urothelial cells. Histochem. Cell Biol..

[67] Hussein N.A., Malla S., Pasternak M.A., Terrero D., Brown N.G., Ashby C.R., Assaraf Y.G., Chen Z.-S., Tiwari A.K. (2021). The role of endolysosomal trafficking in anticancer drug resistance. Drug Resist. Updates.

[68] Prabhakar N., Khan M.H., Peurla M., Chang H.-C., Hänninen P.E., Rosenholm J.M. (2017). Intracellular Trafficking of Fluorescent Nanodiamonds and Regulation of Their Cellular Toxicity. ACS Omega.

[69] Kramer M.W., Waalkes S., Serth J., Hennenlotter J., Tezval H., Stenzl A., Kuczyk M.A., Merseburger A.S. (2011). Decreased galectin-8 is a strong marker for recurrence in urothelial carcinoma of the bladder. Urol. Int..

[70] Przybylo M., Hoja-Lukowicz D., Litynska A., Laidler P. (2002). Different glycosylation of cadherins from human bladder non-malignant and cancer cell lines. Cancer Cell Int..

[71] Przybylo M., Litynska A., Pochec E. (2005). Different adhesion and migration properties of human HCV29 non-malignant urothelial and T24 bladder cancer cells: Role of glycosylation. Biochimie.

[72] Apfelthaler C., Gassenbauer P., Weisse S., Gabor F., Wirth M. (2018). A lectin mediated delivery system for the intravesical treatment of bladder diseases using poly-(L)-glutamic acid as polymeric backbone. Eur. J. Pharm. Sci..

[73] Neutsch L., Eggenreich B., Herwig E., Marchetti-Deschmann M., Allmaier G., Gabor F., Wirth M. (2012). Lectin bioconjugates trigger urothelial cytoinvasion—A glycotargeted approach for improved intravesical drug delivery. Eur. J. Pharm. Biopharm..

[74] Neutsch L., Wirth E.M., Spijker S., Pichl C., Kählig H., Gabor F., Wirth M. (2013). Synergistic targeting/prodrug strategies for intravesical drug delivery–lectin-modified PLGA microparticles enhance cytotoxicity of stearoyl gemcitabine by contact-dependent transfer. J. Control Release.

[75] Tratnjek L., Jeruc J., Romih R., Zupančič D. (2021). Vitamin A and Retinoids in Bladder Cancer Chemoprevention and Treatment: A Narrative Review of Current Evidence, Challenges and Future Prospects. Int. J. Mol. Sci..

[76] Chow E.K., Zhang X.Q., Chen M., Lam R., Robinson E., Huang H., Schaffer D., Osawa E., Goga A., Ho D. (2011). Nanodiamond therapeutic delivery agents mediate enhanced chemoresistant tumor treatment. Sci. Transl. Med..

[77] Man H.B., Kim H., Kim H.-J., Robinson E., Liu W.K., Chow E.K.-H., Ho D. (2014). Synthesis of nanodiamond–daunorubicin conjugates to overcome multidrug chemoresistance in leukemia. Nanomed. Nanotechnol. Biol. Med..

[78] Balmer J.E., Blomhoff R. (2002). Gene expression regulation by retinoic acid. J. Lipid Res..

[79] Zhu G. (2019). Vitamin A and its Derivatives-Retinoic Acid and Retinoid Pharmacology. Am. J. Biomed. Sci. Res..

[80] Shimkunas R.A., Robinson E., Lam R., Lu S., Xu X., Zhang X.Q., Huang H., Osawa E., Ho D. (2009). Nanodiamond-insulin complexes as pH-dependent protein delivery vehicles. Biomaterials.

[81] Xi G., Robinson E., Mania-Farnell B., Vanin E.F., Shim K.W., Takao T., Allender E.V., Mayanil C.S., Soares M.B., Ho D. (2014). Convection-enhanced delivery of nanodiamond drug delivery platforms for intracranial tumor treatment. Nanomedicine.

[82] Li Y., Tong Y., Cao R., Tian Z., Yang B., Yang P. (2014). In vivo enhancement of anticancer therapy using bare or chemotherapeutic drug-bearing nanodiamond particles. Int. J. Nanomed..

[83] Locharoenrat K. (2019). Efficacy of nanodiamond-doxorubicin complexes on human breast adenocarcinoma cell lines. Artif. Cells Nanomed. Biotechnol..

[84] Huang H., Pierstorff E., Osawa E., Ho D. (2007). Active nanodiamond hydrogels for chemotherapeutic delivery. Nano Lett..

[85] Perevedentseva E., Lin Y.C., Cheng C.L. (2021). A review of recent advances in nanodiamond-mediated drug delivery in cancer. Expert Opin. Drug Deliv..

[86] Li T.F., Li K., Zhang Q., Wang C., Yue Y., Chen Z., Yuan S.J., Liu X., Wen Y., Han M. (2018). Dendritic cell-mediated delivery of doxorubicin-polyglycerol-nanodiamond composites elicits enhanced anti-cancer immune response in glioblastoma. Biomaterials.

[87] Alawdi S.H., El-Denshary E.S., Safar M.M., Eidi H., David M.O., Abdel-Wahhab M.A. (2017). Neuroprotective Effect of Nanodiamond in Alzheimer’s Disease Rat Model: A Pivotal Role for Modulating NF-κB and STAT3 Signaling. Mol. Neurobiol..

[88] Dielubanza E.J., Schaeffer A.J. (2011). Urinary tract infections in women. Med. Clin. N. Am..

[89] Bower J.M., Eto D.S., Mulvey M.A. (2005). Covert operations of uropathogenic Escherichia coli within the urinary tract. Traffic.

[90] Xie B., Zhou G., Chan S.Y., Shapiro E., Kong X.P., Wu X.R., Sun T.T., Costello C.E. (2006). Distinct glycan structures of uroplakins Ia and Ib: Structural basis for the selective binding of FimH adhesin to uroplakin Ia. J. Biol. Chem..

[91] Zhou G., Mo W.J., Sebbel P., Min G., Neubert T.A., Glockshuber R., Wu X.R., Sun T.T., Kong X.P. (2001). Uroplakin Ia is the urothelial receptor for uropathogenic *Escherichia coli*: Evidence from in vitro FimH binding. J. Cell Sci..

[92] Wu X.R., Sun T.T., Medina J.J. (1996). In vitro binding of type 1-fimbriated *Escherichia coli* to uroplakins Ia and Ib: Relation to urinary tract infections. Proc. Natl. Acad. Sci. USA.

[93] Klemm P., Hjerrild L., Gjermansen M., Schembri M.A. (2004). Structure-function analysis of the self-recognizing Antigen 43 autotransporter protein from *Escherichia coli*. Mol. Microbiol..

[94] Eto D.S., Sundsbak J.L., Mulvey M.A. (2006). Actin-gated intracellular growth and resurgence of uropathogenic *Escherichia coli*. Cell Microbiol..

[95] Blango M.G., Mulvey M.A. (2010). Persistence of uropathogenic *Escherichia coli* in the face of multiple antibiotics. Antimicrob. Agents Chemother..

[96] Barber A.E., Norton J.P., Spivak A.M., Mulvey M.A. (2013). Urinary tract infections: Current and emerging management strategies. Clin. Infect. Dis..

[97] Hartmann M., Lindhorst T.K. (2011). The Bacterial Lectin FimH, a Target for Drug Discovery—Carbohydrate Inhibitors of Type 1 Fimbriae-Mediated Bacterial Adhesion. Eur. J. Org. Chem..

[98] Barras A., Martin F.A., Bande O., Baumann J.S., Ghigo J.M., Boukherroub R., Beloin C., Siriwardena A., Szunerits S. (2013). Glycan-functionalized diamond nanoparticles as potent *E. coli* anti-adhesives. Nanoscale.

[99] Iyer J.K., Dickey A., Rouhani P., Kaul A., Govindaraju N., Singh R.N., Kaul R. (2018). Nanodiamonds facilitate killing of intracellular uropathogenic *E. coli* in an in vitro model of urinary tract infection pathogenesis. PLoS ONE.

[100] Min G., Zhou G., Schapira M., Sun T.T., Kong X.P. (2003). Structural basis of urothelial permeability barrier function as revealed by Cryo-EM studies of the 16 nm uroplakin particle. J. Cell Sci..

[101] Zaak D., Hungerhuber E., Schneede P., Stepp H., Frimberger D., Corvin S., Schmeller N., Kriegmair M., Hofstetter A., Knuechel R. (2002). Role of 5-aminolevulinic acid in the detection of urothelial premalignant lesions. Cancer.

[102] Mowatt G., N’Dow J., Vale L., Nabi G., Boachie C., Cook J.A., Fraser C., Griffiths T.R. (2011). Photodynamic diagnosis of bladder cancer compared with white light cystoscopy: Systematic review and meta-analysis. Int. J. Technol. Assess. Health Care.

[103] Draga R.O., Grimbergen M.C., Kok E.T., Jonges T.N., Bosch J.L. (2009). Predictors of false positives in 5-aminolevulinic acid-induced photodynamic diagnosis of bladder carcinoma: Identification of patient groups that may benefit most from highly specific optical diagnostics. Urology.

[104] Khanal M., Larsonneur F., Raks V., Barras A., Baumann J.-S., Martin F.A., Boukherroub R., Ghigo J.-M., Ortiz Mellet C., Zaitsev V. (2015). Inhibition of type 1 fimbriae-mediated *Escherichia coli* adhesion and biofilm formation by trimeric cluster thiomannosides conjugated to diamond nanoparticles. Nanoscale.

[105] Lien Z.Y., Hsu T.C., Liu K.K., Liao W.S., Hwang K.C., Chao J.I. (2012). Cancer cell labeling and tracking using fluorescent and magnetic nanodiamond. Biomaterials.

[106] Ni J.S., Li Y., Yue W., Liu B., Li K. (2020). Nanoparticle-based Cell Trackers for Biomedical Applications. Theranostics.

[107] Chen G., Zhang Y., Li C., Huang D., Wang Q., Wang Q. (2018). Recent Advances in Tracking the Transplanted Stem Cells Using Near-Infrared Fluorescent Nanoprobes: Turning from the First to the Second Near-Infrared Window. Adv. Healthc. Mater..

[108] Zhao P., Xu Q., Tao J., Jin Z., Pan Y., Yu C., Yu Z. (2018). Near infrared quantum dots in biomedical applications: Current status and future perspective. Interdiscip. Rev. Nanomed. Nanobiotechnol..

